# Characterization of novel interactions with membrane NEU1 highlights new regulatory functions for the Elastin Receptor Complex in monocyte interaction with endothelial cells

**DOI:** 10.1186/s13578-021-00718-x

**Published:** 2021-12-13

**Authors:** Olivier Bocquet, Dignê Tembely, Damien Rioult, Christine Terryn, Béatrice Romier, Amar Bennasroune, Sébastien Blaise, Hervé Sartelet, Laurent Martiny, Laurent Duca, Pascal Maurice

**Affiliations:** 1 UMR CNRS 7369 Matrice Extracellulaire Et Dynamique Cellulaire (MEDyC), Université de Reims Champagne Ardenne (URCA), UFR Sciences Exactes Et Naturelles, Moulin de La Housse, BP1039, 51687 Reims cedex 2, France; 2 Plateau Technique Mobile de Cytométrie Environnementale MOBICYTE, URCA/INERIS, Université de Reims Champagne Ardenne (URCA), UFR Sciences Exactes Et Naturelles, Reims, France; 3 Plate-Forme Imagerie Cellulaire Et Tissulaire (PICT), Université de Reims Champagne Ardenne (URCA), UFR Médecine, Reims, France

**Keywords:** Elastin remodeling, Elastin peptides, Elastin receptor complex, NEU1, Desialylation, Membrane glycoproteins, Monocytes, Endothelial cells

## Abstract

**Background:**

Vascular aging is associated with remodeling of elastin, one of the main extracellular matrix component of the arterial wall, and production of elastin-derived peptides (EDP). These extracellular matrix degradation products have been shown to trigger biological activities through the elastin receptor complex (ERC) and data from the last decade have brought significant insights on the critical role played by its NEU1 subunit in the biological effects mediated by EDP and the ERC in vascular and metabolic diseases.

**Results:**

Using a proteomic approach, we previously identified new potential interaction partners of membrane NEU1. Here, we validated the interaction between NEU1 and the β_2_ integrin in human monocytes and show that binding of EDP to the ERC leads to desialylation of β_2_ integrin through NEU1. A similar action mechanism was identified in human umbilical vein endothelial cells (HUVEC) for intercellular cell adhesion molecule-1 (ICAM-1). Importantly, these effects were associated with a significant increase in monocyte adhesion to endothelial cells and monocyte transendothelial migration.

**Conclusions:**

These results demonstrate that membrane NEU1 sialidase interacts and modulates the sialylation levels of the β_2_ integrin and ICAM-1 through the ERC in monocytes and endothelial cells, respectively, and suggest that EDP and the ERC, through this newly identified common mode of action governed by NEU1, may be important regulators of circulating monocyte recruitment to inflamed vascular sites. Moreover, by its ability to interact with and to modulate the sialylation of key membrane glycoproteins through NEU1, new biological functions are anticipated for EDP and the ERC in elastin remodeling-associated disorders.

## Background

Vascular remodeling underlies the pathogenesis of most of cardiovascular diseases and involves reorganization and degradation of the vascular extracellular matrix (ECM). Elastin, the major component of elastic fibers, represents one of the main structural matrix components of the vascular wall together with collagens. This highly stable and long-life matrix protein [1] is essential for maintaining the strength, resilience, and integrity of the vessel wall [2]. But beyond this fundamental role, a huge amount of works from the last decade highlighted that elastin plays also a pivotal role in various pathophysiological processes through the release of elastin-derived peptides (EDP), including vascular and metabolic diseases. This has been the subject of several reviews in the field [3–7]. Indeed, low-grade inflammation together with increase in proteolytic activity, non-enzymatic post-translational modifications, and deposition of calcium and lipids, are typical features of pathophysiological vascular aging that contribute to elastic fibers and elastin degradation [3, 7, 8]. Elastin degradation releases EDP, also known as elastokines, that trigger biological activities. Although several receptors may mediate the biological effects of EDP, such as the galectin-3 receptor [9], α_v_β_3_ and α_v_β_5_ integrins [10, 11] and a lactose-insensitive elastin receptor [12], most of their biological effects have been attributed so far to the elastin receptor complex (ERC). This membrane receptor brings together the elastin binding protein (EBP), protective protein/cathepsin A (PPCA) and neuraminidase-1 (NEU1) [13].

NEU1 is part of the sialidase family that are exoglycosidases removing terminal sialic acid residues from glycoproteins, glycolipids and oligosaccharides. Initially described as a lysosomal sialidase, NEU1 is also expressed at the plasma membrane where it regulates a myriad of glycoproteins by desialylation, such as integrins [14, 15], receptor tyrosine kinases [16–18] and Toll-like receptors [19, 20], resulting in modulation of receptor activation and signaling. Moreover, in complex with MMP-9, G-protein coupled receptors, and receptor tyrosine kinases or Toll-like receptors, NEU1 would be involved in receptor transactivation [21, 22]. By its ability to interact with different membrane glycoproteins and to modulate their sialylation levels, NEU1 was shown to be involved in several biological processes such as cell migration, proliferation, invasion and adhesion, potentiation of exocytosis [23], and thereby, in a wide range of human disorders, including neurodegenerative disorders, cancers, infectious and cardiovascular diseases [24]. At the plasma membrane, NEU1 also constitutes the signal transducing subunit of the ERC [25], and is considered as a crucial regulator of elastic fibers homeostasis through its involvement in elastic fibers assembly and degradation sensing, and likely in the regulation of Transforming Growth Factor β bioavailability [26]. NEU1 plays a pivotal role in the biological effects mediated by EDP and the ERC in various vascular and metabolic diseases [16, 27–29]. Together, these recent findings make NEU1 a pharmacological target of high added-value and the identification and functional characterization of novel protein interactions with this plasma membrane sialidase will help to highlight new roles played by NEU1 in the biological processes mediated by EDP and the ERC [26]. In the search for new interacting partners of membrane NEU1 in human macrophages, we recently developed a proteomic approach based on a two-step purification of membrane NEU1 and its associated protein complexes [30]. Among the different protein candidates, we previously reported, for the first time, the presence of CD36. We showed that binding of EDP to the ERC induced desialylation of CD36 through NEU1, leading to potentiation of oxidized LDL uptake in macrophages [30] that may contribute to the previously reported pro-atherogenic effects of EDP [27]. Among the potential candidates, we also identified the β_2_ integrin [30].

In the present study, we validated the interaction with membrane NEU1 and β_2_ integrin in human monocytes and report that binding of EDP to the ERC leads to β_2_ integrin desialylation through membrane NEU1 in these cells. A similar mode of action was observed in human umbilical vein endothelial cells (HUVEC) for intercellular cell adhesion molecule-1 (ICAM-1). Importantly, desialylation of either monocyte β_2_ integrin or endothelial ICAM-1 is sufficient to potentiate monocyte adhesion to a monolayer of endothelial cells that is also associated with increase in monocyte transendothelial migration. Together, these results demonstrate, for the first time, that the ERC interacts with β_2_ integrin and ICAM-1 through membrane NEU1 in monocytes and endothelial cells, respectively, and that binding of EDP to the ERC modulates β_2_ integrin and ICAM-1 sialylation levels through this sialidase. These effects are associated with a significant increase in monocyte adhesion to endothelial cells and in monocyte transendothelial migration. By its ability to interact with and to modulate the sialylation of key membrane glycoproteins via NEU1, and through this newly identified common mode of action, new biological functions are anticipated for the ERC in diseases involving elastic fibers and elastin remodeling and degradation.

## Results

We previously developed a proteomic approach dedicated to the purification and identification of membrane NEU1-associated protein complexes in human macrophages [30]. This two-step purification approach uses biotinylation of plasma membrane proteins, immobilization of biotinylated membrane proteins to monomeric avidin beads followed by immunoprecipitation of NEU1 and its associated protein complexes after elution of bound proteins to avidin beads by free biotin. Immunoprecipitated NEU1 and its protein complexes were then fractionated by SDS-PAGE, excised from the gel for protein identification by mass spectrometry. Among the potential candidates, the β_2_ integrin was identified from 36 unique peptides, corresponding to an amino acid sequence coverage of 44% [30].

### Membrane NEU1 interacts with β_2_ integrin and modulates its sialylation level in human monocytes following EDP stimulation

**Fig. 1:**
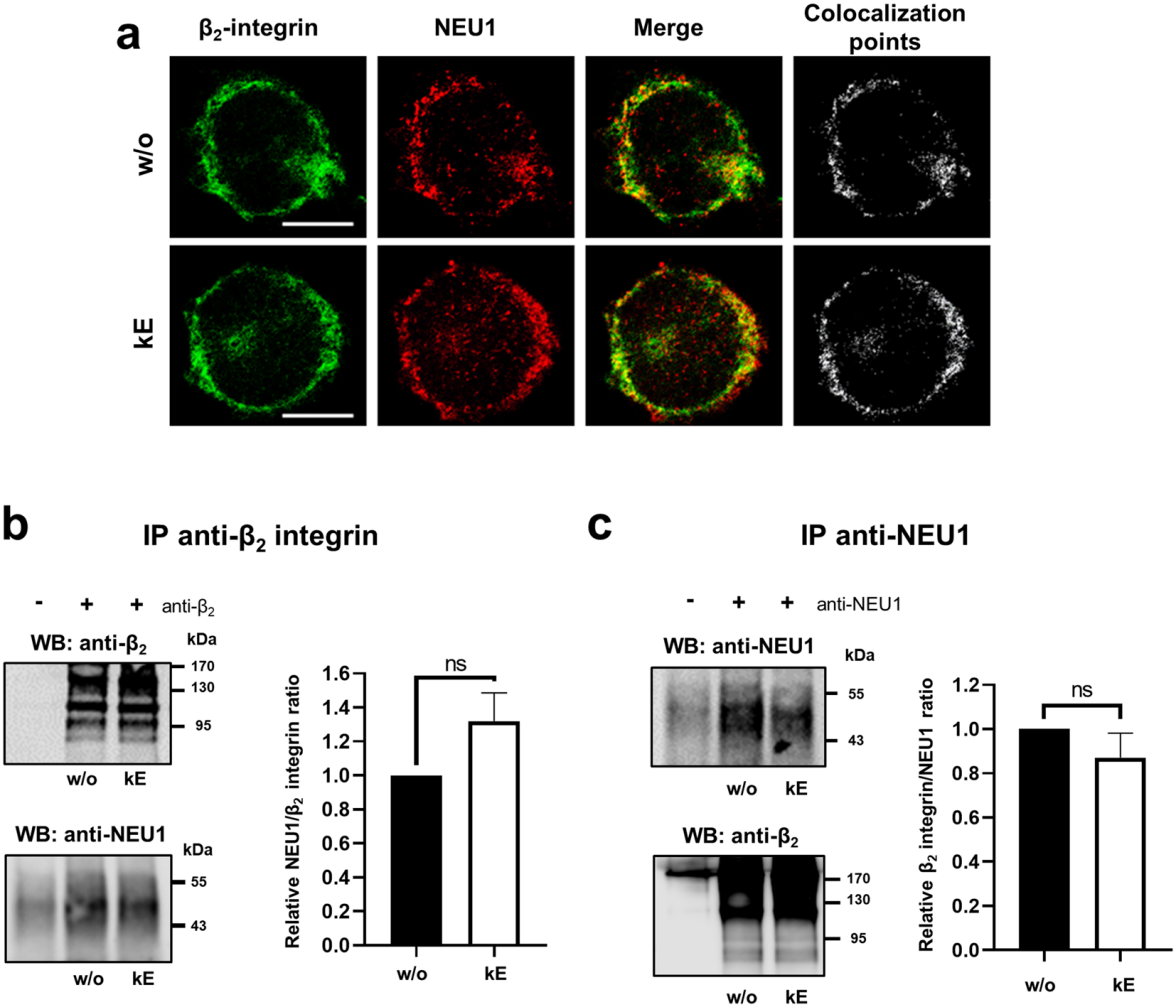
NEU1 interacts with β_2_ integrin in human monocytes. **a** Colocalization between NEU1 and β_2_ integrin at the cell surface of THP-1 monocytes stimulated, or not, by κE (50 µg/mL, 1 h) by confocal microscopy. Areas of colocalization at the plasma membrane were analyzed by ImageJ software and are indicated in white on the right panels. Scale bar: 10 µm. **b** Left panel, β_2_ integrin was immunoprecipitated with a mouse monoclonal anti-β_2_ integrin from whole lysates of THP-1 cells and co-immunoprecipitation of NEU1 was monitored by Western blot. The image is representative of 6 independent experiments. Right panel, blot quantification by densitometry analysis. Results are expressed as mean ± SEM of 6 independent experiments and normalized to the basal condition (without κE, w/o). Statistical analysis was performed by Student’s *t*-test (ns, non-significant). **c** NEU1 was immunoprecipitated with a mouse monoclonal anti-NEU1 antibody from whole lysates of THP-1 cells and co-immunoprecipitation of β_2_ integrin was monitored by Western blot. The image is representative of 6 independent experiments. Right panel: blot quantification by densitometry analysis. Results are expressed as mean ± SEM of 6 independent experiments and normalized to the basal condition (w/o). Statistical analysis was performed by Student’s *t*-test (ns, non-significant)

The localization of NEU1 and the β_2_ integrin was first evaluated in the human monocytic THP-1 cell line. As shown in Fig. 1a, the β_2_ integrin mainly localized at the plasma membrane and NEU1 was detected both intracellularly and at the plasma membrane. Merging both acquisitions signals clearly showed colocalization of NEU1 and the β_2_ integrin at the plasma membrane of human monocytes, and prior stimulation of the cells by EDP, derived from organo-alkaline hydrolysate of insoluble bovine purified elastin (kappa-elastin, κE), did not modify their co-localization. Interaction between NEU1 and β_2_ integrin was confirmed by co-immunoprecipitation experiments. Immunoprecipitation of β_2_ integrin in non-reduced conditions revealed the presence of three major protein bands ranging from 100 to 150 kDa in THP-1 monocytes, likely due to the presence of several N-linked glycosylation sites and potential disulphide bridges within the β_2_ integrin (Fig. 1b). Immunoprecipitation of β_2_ integrin was shown to co-immunoprecipitate NEU1. A trend for increase in co-immunoprecipitation of NEU1 with the β_2_ integrin, that failed to be significant (*p* = 0.0821), was observed after stimulation of the cells with κE (50 µg/mL). Reciprocally, immunoprecipitation of NEU1 also co-immunoprecipitated the β_2_ integrin and no further increase was observed after stimulation by EDP (Fig. 1c). Together, these data showed that NEU1 and β_2_ integrin constitutively interacted in human monocytes.

**Fig. 2:**
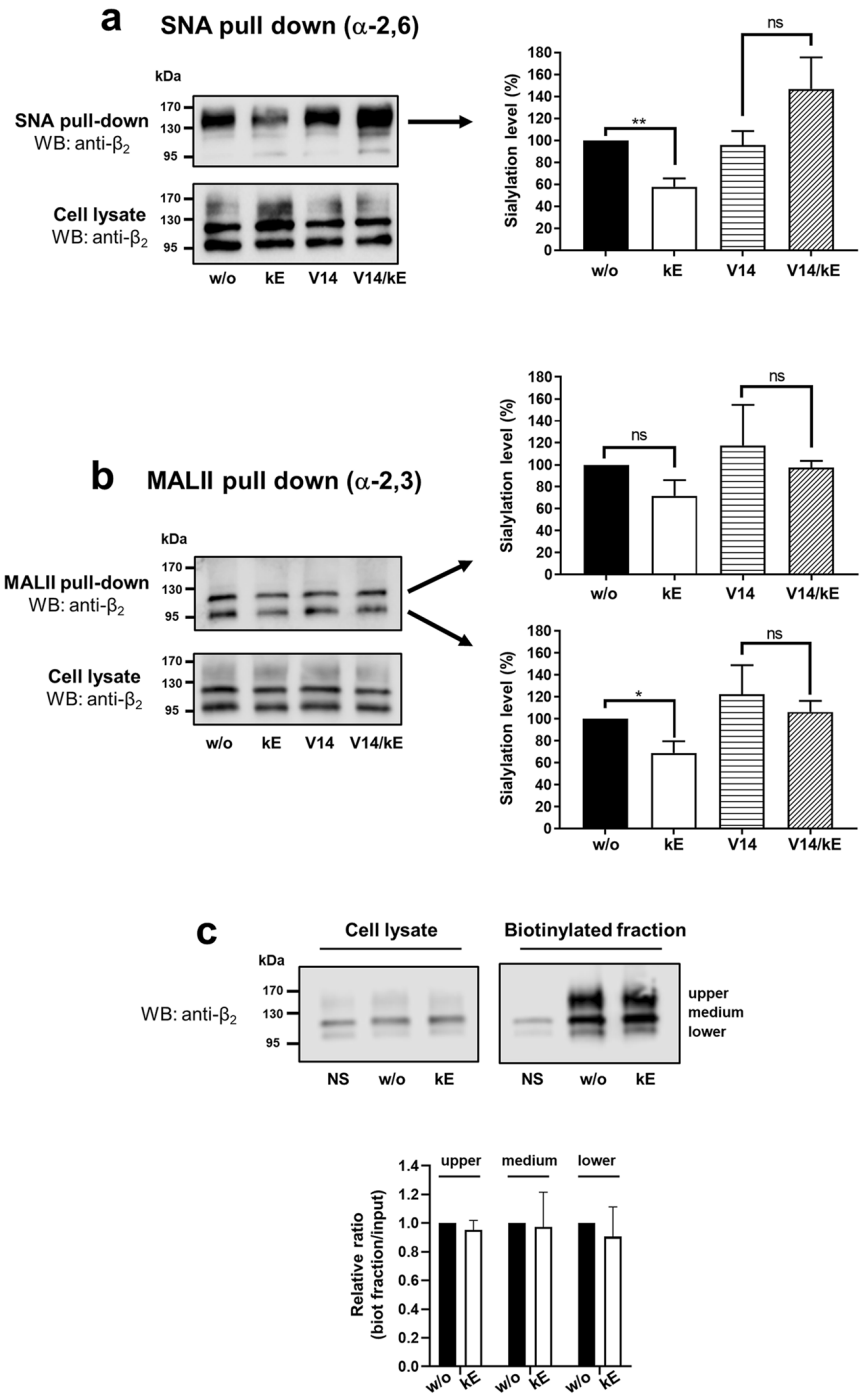
Binding of EDP to the ERC decreases the sialylation level of β_2_ integrin through NEU1 in human monocytes. **a**, **b** Left panels: SNA and MALII pull down of crude membrane preparations of THP-1 cells incubated, or not, with κE (50 µg/mL), V14 + κE (molar ratio 2:1) or V14 peptide alone for 1 h at 37 °C. For each condition, equal amount of proteins was used. The amount of sialylated β_2_ integrin in α-2,6 (SNA) or α-2,3 (MALII) recovered after lectin pull down was evaluated by Western blot using a mouse monoclonal anti-β_2_ integrin antibody. The image is representative of 4 independent experiments. Right panels: quantification of α-2,6 and or α-2,3 sialylation levels of β_2_ integrin (pull down/lysate ratio) by densitometry analysis, and normalized to the basal condition (without κE, w/o). Results are expressed as mean ± SEM of 4 independent experiments and statistical analysis was performed by Student’s *t*-test (*p < 0.05; **, *p* < 0.01; ns, non-significant). **c** Top, western blot on the biotinylated and cell lysate fractions of THP-1 cells stimulated, or not, with κE (50 µg/mL, 1 h), and probed with a mouse monoclonal anti-β_2_ integrin antibody. A representative pattern is shown. The NS lane corresponds to non-biotinylated cells used for estimation of non-specific binding of β_2_ integrin to streptavidin beads. Bottom, quantification of the relative expression of β_2_ integrin in the biotinylated fraction by densitometry analysis. Relative expression was calculated as amount of β_2_ integrin recovered in the biotinylated fraction over expression level in cell lysate and normalized to the basal condition (w/o). Results are expressed as mean ± SEM of 3 independent experiments

We next assessed the functional relevance of this interaction by evaluating the effects of EDP on α-2,6 and α-2,3 sialylation levels of β_2_ integrin using a lectin pull down assay and the *Sambucus nigra* Agglutinin (SNA) and *Maackia amurensis* lectin II (MALII), respectively (Fig. 2). As previously reported, κE stimulation of THP1-derived macrophages triggers membrane sialidase activity that is dependent on NEU1 [30]. The three protein bands detected by the anti-β_2_ integrin antibody were shown to be differentially sialylated at resting state; the higher band (~ 150 kDa) being mostly α-2,6 sialylated (Fig. 2a) and the two lower bands (~ 100 kDa, ~ 120 kDa) being mainly α-2,3 sialylated (Fig. 2b). Interestingly, stimulation of monocytes by κE (50 µg/mL) was associated with a significant decrease by 42.1 ± 7.7% of the sialylation level of the ~ 150 kDa protein and by 31.1 ± 10.6% for the ~ 100 kDa protein. A trend for a decrease of the sialylation level of the ~ 120 kDa protein, that failed to be significant (*p* = 0.0730), was also observed.

To confirm that this modulating effect on β_2_ integrin sialylation level involved the ERC, κE was pre-incubated with the V14 peptide (VVGSPSAQDEASPL), a synthetic peptide blocking the interaction between the GxxPG motifs contained in the κE mixture and the ERC [31, 32], prior to its incubation with THP-1 monocytes. In these conditions, the effects of κE were blocked (Fig. 2a, b). Together with our previous data pointing out that membrane sialidase activity triggered by κE was blocked by NEU1 siRNA in THP1-derived macrophages [30], these results demonstrated that both the ERC and its NEU1 subunit were involved in these modulating effects of EDP. Of note, decrease of β_2_ integrin sialylation level by EDP was not associated with modulation of cell surface expression levels of the integrin (Fig. 2c).

### Modulation of β_2_ integrin sialylation level through NEU1 increases adhesion of monocytes to human endothelial cells

Together with CD11a or CD11b, β_2_ integrin (CD18) forms the LFA-1 and Mac-1 adhesion complexes that are receptors for ICAM-1, also known as CD54 [33], a surface molecule which is constitutively expressed at low levels on vascular endothelial cells and on some lymphocytes and monocytes [34]. Of note, ICAM-1 was also recovered in our proteomic screen as potential interaction partner of membrane NEU1 (from 25 unique peptides, corresponding to an amino acid sequence coverage of 46%). Expression of ICAM-1 is known to be increased upon stimulation by inflammatory cytokines, lipopolysaccharide or phorbol esters such as phorbol myristate acetate (PMA) [35]. As a ligand for β_2_ integrins present on leukocytes, ICAM-1 expressed by endothelial cells participates in leukocyte rolling, adhesion and transmigration across the endothelium [36]. This process is fundamental to inflammatory diseases, including atherosclerosis, where monocytes enter into the intimal space to form the foam cells and fatty streaks. We therefore investigated if EDP-induced changes in the sialylation levels of the β_2_ integrin may affect monocyte adhesion onto a monolayer of endothelial cells.

**Fig. 3:**
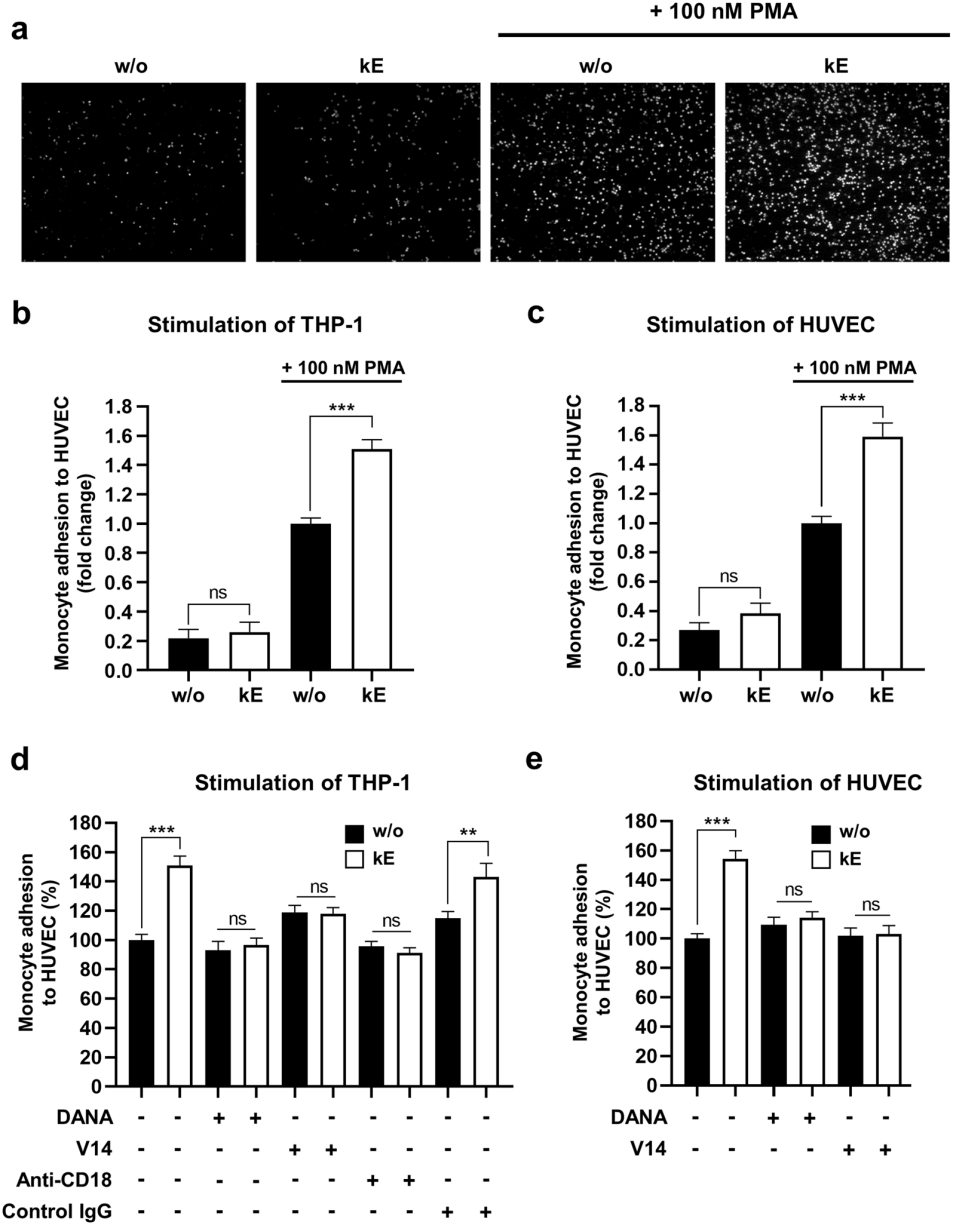
Binding of EDP to the ERC potentiates monocyte adhesion to endothelial cells through NEU1. (**a, b**) THP-1 monocytes were incubated, or not, with κE (50 µg/mL, 1 h), labelled with Calcein-AM, and allowed to adhere for 30 min at 37 °C onto a monolayer of HUVEC pre-stimulated, or not, with PMA (100 nM). Cells were then washed, fixed and visualized under a fluorescent microscope. **a** A representative field is shown. **b** The percentage of surface area covered by adherent monocytes was calculated for each condition from 10 different fields per experiment, each run in duplicate. Results were expressed as mean ± SEM of 4–8 independent experiments and normalized to the control condition (without, w/o) in presence of PMA. Statistical analysis was performed by Student’s *t*-test (***p < 0.001; ns: non-significant). **c** Similar experiments as in **b** except that HUVEC, instead of THP-1 cells, were incubated, or not, with κE (50 µg/mL, 1 h) prior to monocyte adhesion. Results were expressed as mean ± SEM of 3–4 independent experiments. Statistical analysis was performed by Student’s *t*-test (***p < 0.001; ns: non-significant). **d** THP-1 monocytes were incubated, or not, with DANA (400 µM), anti-CD18 blocking antibody (10 µg/mL) or isotype control (10 µg/mL) for 1 h at 37 °C before stimulation, or not, by κE (50 µg/mL), V14 + κE (molar ratio 2:1) or V14 peptide alone for 1 h at 37 °C. After adhesion (30 min, 37 °C), the percentage of surface area covered by adherent monocytes was calculated for each condition from 10 different fields per experiment, each run in duplicate. Results were expressed as mean ± SEM of 3–4 independent experiments, and normalized to the control condition (w/o). Statistical analysis was performed by Student’s *t*-test (**p < 0.01; ***p < 0.001; ns: non-significant). Black bars, non-stimulated cells; white bars, κE-stimulated cells. **e** Similar experiments as in **d** except that HUVEC, instead of THP-1 cells, were incubated, or not, with DANA (400 µM) followed by stimulation, or not, κE (50 µg/mL), V14 + κE (molar ratio 2:1) or V14 peptide alone for 1 h at 37 °C. Results were expressed as mean ± SEM of 3–6 independent experiments. Statistical analysis was performed by Student’s *t*-test (***p < 0.001; ns: non-significant)

**Fig. 4:**
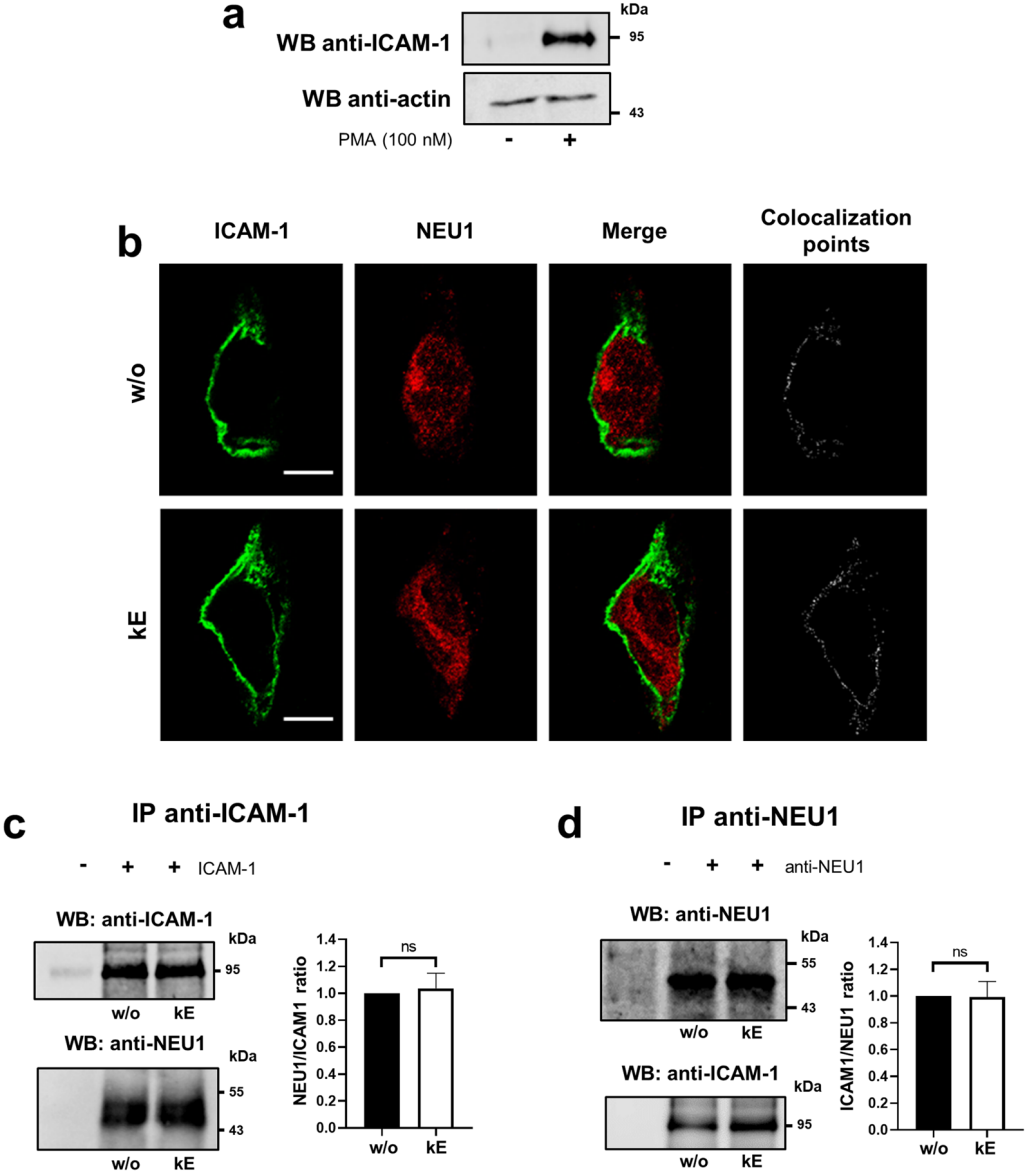
NEU1 interacts with ICAM-1 in human endothelial cells. **a** Detection of ICAM-1 expression by Western blot in whole lysate of HUVEC pre-stimulated, or not, with PMA (100 nM, overnight). **b** Colocalization between NEU1 and ICAM-1 at the cell surface of PMA-pre-stimulated HUVEC incubated, or not, with κE (50 µg/mL, 1 h) by confocal microscopy. Areas of colocalization at the plasma membrane were analyzed by ImageJ software and are indicated in white on the right panels. Scale bar: 10 µm. **c** Left panel, ICAM-1 was immunoprecipitated with a mouse monoclonal anti-ICAM-1 antibody from whole lysates of PMA-pre-stimulated HUVEC and co-immunoprecipitation of NEU1 was monitored by Western blot. The image is representative of 3 independent experiments. Right panel, blot quantification by densitometry analysis. Results are expressed as mean ± SEM of 3 independent experiments and normalized to the basal condition (without κE, w/o). Statistical analysis was performed by Student’s *t*-test (ns, non-significant). **d** NEU1 was immunoprecipitated with a mouse monoclonal anti-NEU1 antibody from whole lysates of PMA-pre-stimulated HUVEC and co-immunoprecipitation of ICAM-1 was monitored by Western blot. The image is representative of 3 independent experiments. Right panel: blot quantification by densitometry analysis. Results are expressed as mean ± SEM of 3 independent experiments and normalized to the basal condition (w/o). Statistical analysis was performed by Student’s *t*-test (ns, non-significant)

Prior to that, we checked the expression level of ICAM-1 in HUVEC and found that ICAM-1 was barely detectable in unstimulated HUVEC (Fig. 4a). In contrast, stimulation of HUVEC by 100 nM PMA (overnight, 37 °C) led to a significant increase in expression of ICAM-1 at the protein level (Fig. 4a) that is associated with a significant increase by fivefold of monocyte adhesion (Fig. 3a). Interestingly, we observed that prior stimulation of monocytes with κE (50 µg/mL) led to a significant increase by 50.8 ± 6.5% of monocyte adhesion to HUVEC. These potentiating effects of EDP were not present when monocytes were allowed to adhere on unstimulated endothelial cells. When using the V14 peptide or 2-deoxy-2,3-didehydro N-acetylneuraminic acid (DANA), a broad-spectrum sialidase inhibitor, these potentiating effects of EDP were blocked demonstrating that both the ERC and the sialidase activity of NEU1 were involved in these effects. Involvement of monocyte β_2_ integrin in EDP-stimulating effects on monocyte adhesion was confirmed by the use of a β_2_ integrin blocking antibody. As shown in Fig. 3d, the use of a blocking antibody against β_2_ integrin had no effect on monocyte adhesion to HUVEC in the absence of kE, suggesting that other monocyte adhesive receptors are likely involved. However, data also showed that, in contrast to a control isotype, this β_2_ integrin blocking antibody totally inhibited the potentiating effects of kE on monocyte adhesion to HUVEC demonstrating that monocyte β2 integrin mediates EDP-stimulated monocyte adhesion to HUVEC.

### Membrane NEU1 also interacts with endothelial ICAM-1 and modulates its sialylation level following EDP stimulation

In PMA-pre-stimulated HUVEC, ICAM-1 was mainly expressed at the plasma membrane and NEU1 mainly detected intracellularly (Fig. 4b). However, discrete NEU1 spots were also observed at the plasma membrane that colocalize with ICAM-1. As well as in monocytes, prior stimulation of cells by EDP did not affect colocalization between NEU1 and ICAM-1 at the plasma membrane. Interaction between NEU1 and ICAM-1 was then evaluated by co-immunoprecipitation. As shown in Fig. 4c, d, immunoprecipitation of ICAM-1 co-immunoprecipitated NEU1 and reciprocally, immunoprecipitation of NEU1 co-immunoprecipitated ICAM-1. Prior stimulation of the cells by κE (50 µg/mL) had no further effect. Together, these results demonstrated that in endothelial cells, NEU1 and ICAM-1 constitutively interact together.

**Fig. 5:**
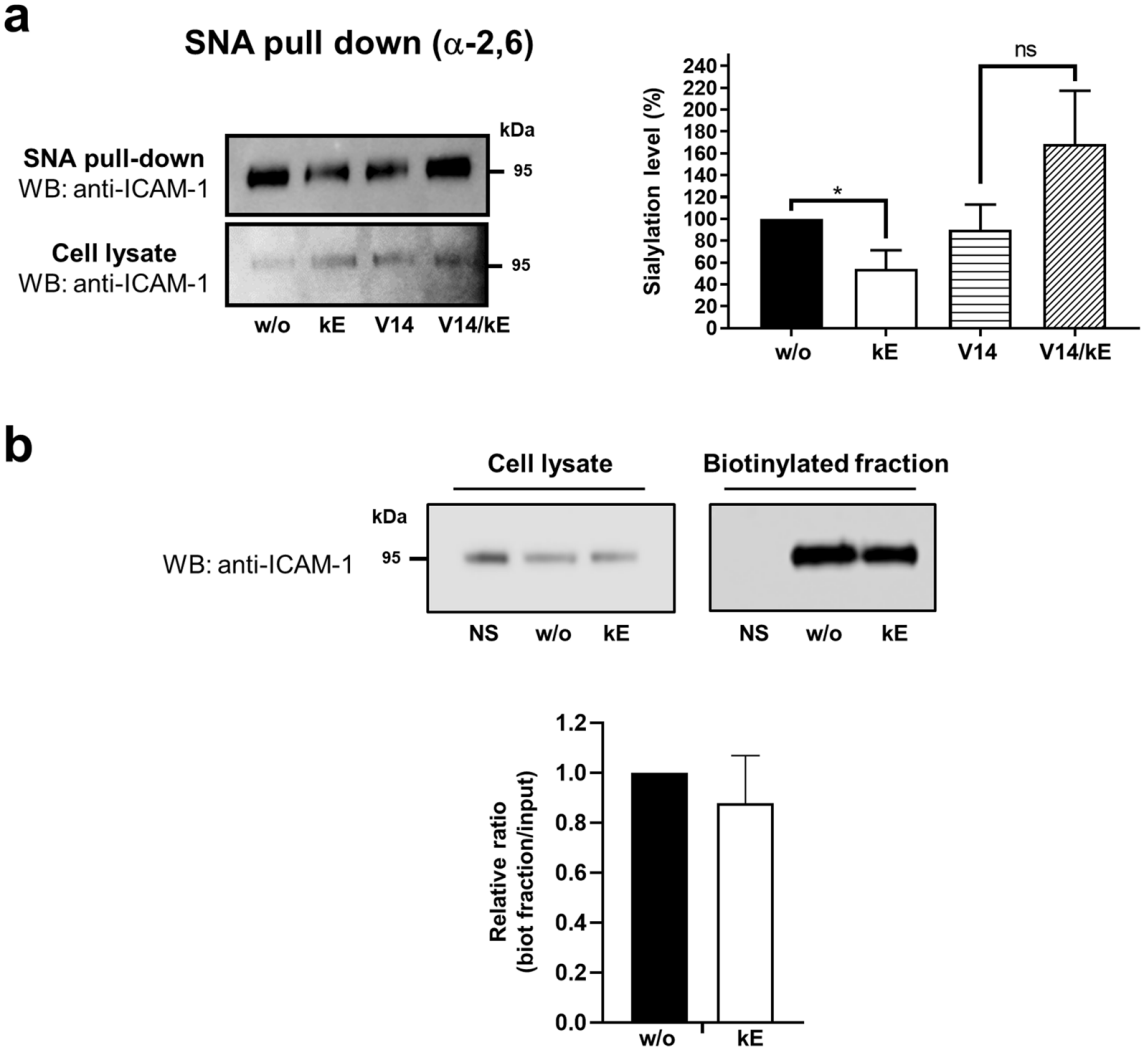
Binding of EDP to the ERC decreases the sialylation level of ICAM-1 through NEU1 in human endothelial cells. **a** Left panel: SNA pull down of crude membrane preparations of PMA-pre-stimulated HUVEC incubated, or not, with κE (50 µg/mL), V14 + κE (molar ratio 2:1) or V14 peptide alone for 1 h at 37 °C. For each condition, equal amount of proteins was used. The amount of sialylated ICAM-1 recovered after lectin pull down was evaluated by Western blot using a mouse monoclonal anti-ICAM-1 antibody. The image is representative of 3 independent experiments. Right panel: quantification of α-2,6 sialylation level of ICAM-1 (pull down/lysate ratio) by densitometry analysis, and normalized to the basal condition (without κE, w/o). Results are expressed as mean ± SEM of 3 independent experiments and statistical analysis was performed by Student’s *t*-test (*p < 0.05; ns, non-significant). **b** Top, western blot on the biotinylated and cell lysate fractions of PMA-pre-stimulated HUVEC incubated, or not, with κE (50 µg/mL, 1 h), and probed with a mouse monoclonal anti-ICAM-1 antibody. A representative pattern is shown. The NS lane corresponds to non-biotinylated cells used for estimation of non-specific binding of ICAM-1 to streptavidin beads. Bottom, quantification of the relative expression of ICAM-1 in the biotinylated fraction by densitometry analysis. Relative expression was calculated as amount of ICAM-1 recovered in the biotinylated fraction over expression level in cell lysate and normalized to the basal condition (w/o). Results are expressed as mean ± SEM of 3 independent experiments

We next evaluated the functional relevance of this interaction by evaluating the effects of EDP on α-2,6 sialylation level of ICAM-1 by lectin pull down. In HUVEC, ICAM-1 is mainly α-2,6 sialylated with a near-absence of α-2,3 sialylation [37]. As depicted in Fig. 5a, endothelial ICAM-1 harbored α-2,6 sialylation in basal conditions and stimulation of HUVEC by κE (50 µg/mL) was associated with a significant decrease by 46.0 ± 17.5% of the sialylation level of ICAM-1. When κE was pre-incubated with the V14 peptide prior to its incubation with cells, the effects of κE were blocked. Rather, a trend for an increase was observed. Even if the difference failed to be significant, further experiments are required to better understand these unexpected results obtained with the V14/κE mixture. As for β_2_ integrin in monocytes, decrease in ICAM-1 sialylation level was not associated with modulation of its plasma membrane expression level in HUVEC (Fig. 5c). Together, these data show that NEU1 interacts with ICAM-1 in endothelial cells and that binding of EDP to the ERC decreases the sialylation level of ICAM-1 through its NEU1 subunit without affecting it expression level at the plasma membrane.

### Modulation of endothelial ICAM-1 sialylation level through NEU1 is associated with increased monocyte adhesion to HUVEC

**Fig. 6:**
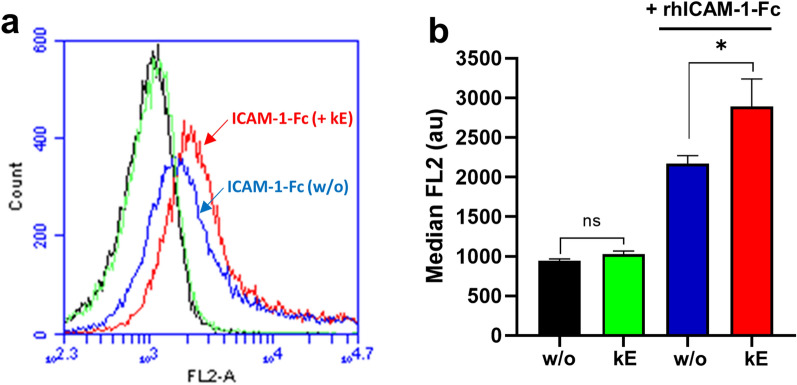
Binding of EDP to the ERC increases ICAM-1 binding on monocytes. **a** THP-1 monocytes (1 × 10^6^/mL) were stimulated, or not, with κE (50 µg/mL) for 1 h at 37 °C, and incubated, or not, with rhICAM-1-Fc/PE-labeled anti-Fc complex for 30 min at room temperature. Data shown are representative of 5 independent experiments. Black trace, control cells; green trace, κE-stimulated cells; blue trace, control cells incubated with rhICAM-1-Fc; red trace, κE-stimulated cells incubated with rhICAM-1-Fc. **b** The graph shows the median fluorescence expressed as mean ± SEM of 5 independent experiments and statistical analysis was performed by Student’s t-test (*p < 0.05; ns, non-significant)

In order to evaluate if decrease in ICAM-1 sialylation level in HUVEC is also associated with modulation of monocyte adhesion to these cells, PMA-pre-stimulated HUVEC were incubated by κE (50 µg/mL, 1 h), washed to remove excess of EDP, and monocyte adhesion was measured as described above. Interestingly, a significant increase by 54.1 ± 7.7% of monocyte adhesion to HUVEC was observed compared to unstimulated HUVEC (Fig. 3c). When using the V14 peptide that blocks the interaction between EDP and the ERC or the broad-spectrum sialidase inhibitor DANA, the potentiating effects of EDP were blocked (Fig. 3e). To prove that these modulating effects involved endothelial ICAM-1, we evaluated the effects of anti-ICAM-1 blocking antibodies. However, in contrast to anti-β_2_ integrin blocking antibodies, pre-incubation of HUVEC with anti-ICAM-1 blocking antibodies led to an unexpected increase in monocyte adhesion of about twofold for the control condition (not shown). We therefore evaluated whether decrease in monocyte β_2_ integrin sialylation level following EDP stimulation was associated with increased ICAM-1 binding to THP-1 monocytes. As shown in Fig. 6, stimulation of monocytes by κE (50 µg/mL, 1 h) induced a significant increase in ICAM-1 binding on monocytes compared to non-stimulated cells (median LF2 intensity from 2168 ± 102 to 2893 ± 347).

### Modulation of either monocyte β_2_ integrin or endothelial ICAM-1 sialylation level through NEU1 following EDP binding to the ERC increases monocyte transendothelial migration

**Fig. 7:**
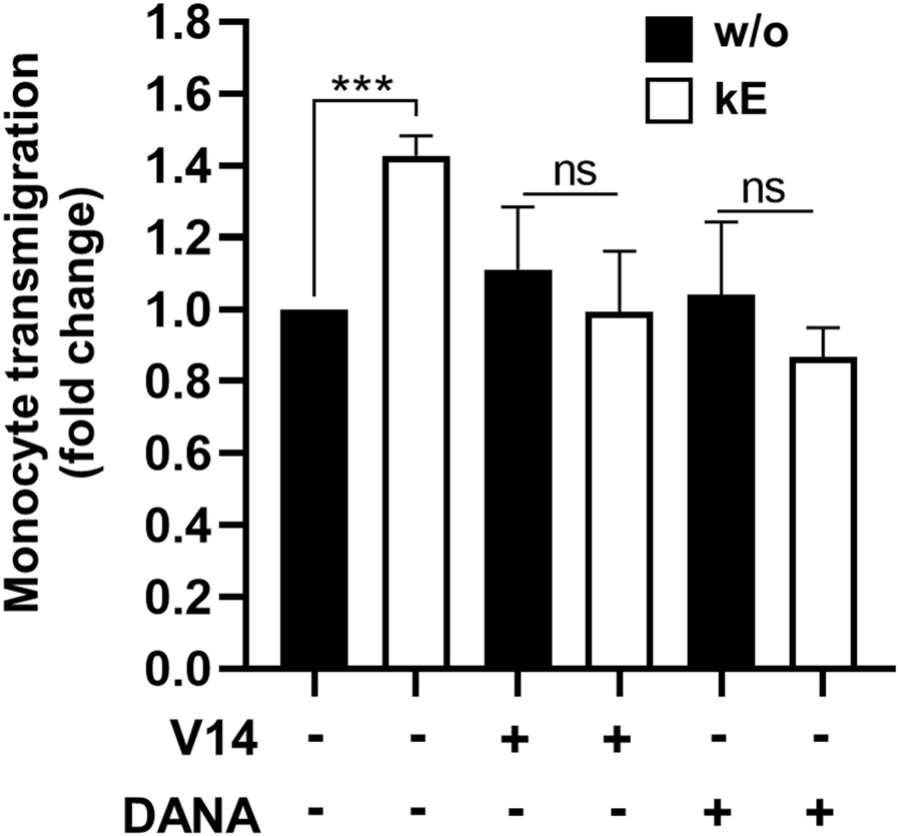
Binding of EDP to the ERC potentiates monocyte transendothelial migration through NEU1. PMA-pre-stimulated HUVEC monolayers in transwells were incubated, or not, with DANA (400 µM) for 1 h at 37 °C before stimulation, or not, by κE (50 µg/mL), V14 + κE (molar ratio 2:1) or V14 peptide alone for 1 h at 37 °C. Calcein AM-labeled THP-1 monocytes were added to the upper chamber and allowed to migrate through the HUVEC monolayer into the lower chamber for 2 h at 37 °C. The upper chamber was then removed and the fluorescence intensity in the lower compartment was measured. Results were expressed as mean ± SEM of 3–4 independent experiments, each run in triplicate, and normalized to the control condition (without, w/o). Statistical analysis was performed by Student’s *t*-test (***p < 0.001; ns: non-significant)

The above data showing that modulation of either monocyte β_2_ integrin or endothelial ICAM-1 sialylation level through NEU1 following EDP binding to the ERC potentiates monocyte adhesion to a monolayer of HUVEC, and that stimulation of monocytes by EDP enhances ICAM-1 binding on monocytes, we finally examined if these potentiating effects may be associated with increase in monocyte transendothelial migration. For this purpose, monocytes were added to the upper chamber of transwells containing PMA-pre-stimulated HUVEC monolayers, and the incubation was conducted for 2 h. When HUVEC were stimulated with κE (50 µg/mL, 1 h), the ability of monocytes for transendothelial migration was significantly enhanced by 42.8 ± 5.6% compared to non-stimulated HUVEC (Fig. 7). In this context, the V14 peptide and DANA totally blocked this potentiating effect of κE. Here again, when monocytes were stimulated by κE, similar effects were observed with a significant increase in monocyte transendothelial migration by 32.6 ± 7.9% compared to non-stimulated monocytes (not shown).

## Discussion

In the present study, we proceeded to the validation and functional characterization of two novel protein interactions with membrane NEU1 taking place in human monocytes for NEU1 and β_2_ integrin interaction, and endothelial cells for NEU1 and ICAM-1 interaction. Both partners were shown to colocalize with NEU1 at the plasma membrane and constitutive interactions were confirmed by co-immunoprecipitations. More importantly, we reported here a common action mechanism for both interactions that involved modulation of β_2_ integrin and ICAM-1 sialylation levels through NEU1 following EDP binding to the ERC. This was attested by the complete abrogation of these modulating effects when using the V14 peptide that blocks the interaction between the GxxPG motifs contained in elastokines and the ERC [31, 32] and the sialidase inhibitor DANA. DANA being a broad-spectrum sialidase inhibitor, one may argue that NEU3, the second membrane sialidase, could be involved in these effects.

However, a large number of studies, based on fibroblasts derived from patients with congenital sialidosis or galactosialidosis, NEU1-deficient mice, mice devoid of NEU1 activity in their hematopoietic lineage, or cells transfected with either NEU1 siRNA or a catalytically inactive NEU1 mutant, has definitely proved that NEU1 is the only membrane sialidase that is part of the ERC and which  plays pivotal role in ERC-mediated signal transduction and biological effects [13, 25, 27, 38]. Recently, another proof was brought by Kawecki et al. reporting that EDP-induced increase of membrane sialidase activity in THP-1-derived macrophages was totally blocked by NEU1 siRNA [30]. In addition, NEU3 is known to require a hydrophobic aglycone, which makes it active mainly towards gangliosides. In contrast, NEU1 is active primarily against sialylated glycopeptides and oligosaccharides with negligible activity against gangliosides [39]. Taken together, all these results have directly and/or indirectly proved that NEU1 is the only membrane sialidase that is linked to the ERC.

Thus, by their ability to increase the catalytic activity of membrane NEU1, EDP binding to the ERC may regulate the sialylation levels of crucial membrane glycoproteins closed to NEU1 within the plasma membrane, anticipating new regulating functions to be discovered for these elastokines. This is of crucial interest with regard to the growing list of membrane receptors that can be regulated by desialylation through NEU1. Desialylation is a pivotal part of sialic acid metabolism, and removing terminal sialic acid residues on glycoproteins, as well as on glycolipids and oligosaccharides, has been shown to modulate the molecular properties and structure of these glycoconjugates, thereby modifying their functions and interactions with other molecules. Whereas the functions of sialic acids have been well recognized, the functional and biological consequences of desialylation processes are still underappreciated. However, accumulating evidence demonstrates that desialylation plays an important role in a variety of pathophysiological processes such as neurodegenerative disorders, cancers, infectious and cardiovascular diseases [24]. The most striking recent example is probably the critical role played by these desialylation processes in platelet clearance [40]. Indeed, when desialylated, platelets are recognized by and bind to the hepatic Ashwell-Morell receptors (AMR) to be removed. Importantly, binding of desialylated platelets to AMR induces hepatic expression of thrombopoietin, thereby regulating platelet production. This recently identified physiological feedback mechanism had major outgrowths for the pathophysiology of platelet diseases, such as essential thrombocythemia and immune thrombocytopenia.

In the present study, we also showed that NEU1-mediated desialylation of either monocyte β_2_ integrin or endothelial ICAM-1 following binding of EDP to the ERC was sufficient to potentiate monocyte adhesion to endothelial cells. Monocyte adhesion to inflamed endothelial cells is a key early event in atherosclerosis formation. By binding to endothelial cells, monocytes then enter the intima by transendothelial migration and differentiate into macrophages to promote fatty streaks formation. Importantly, the results presented here also showed increased ability of monocytes for transendothelial migration following stimulation of both cell types by EDP. Here again, these effects were blocked by the V14 peptide and DANA, strengthening the key role played by the ERC through the catalytic activity of its NEU1 subunit in these events. In addition to monocyte β_2_ integrin and endothelial ICAM-1, other membrane glycoproteins are involved in monocyte adhesion and trafficking across endothelial cells such as the β_1_ integrin, other members of the immunoglobulin superfamily (ICAM-2, PECAM-1, JAM proteins), selectins, ephrins, VE-cadherin, chemokine receptors [41]. Whether the sialylation level of these additional membrane glycoproteins may be modulated by NEU1 following EDP binding to the ERC and in which extent this could participate in the potentiating effects reported here for EDP, remain to be further investigated. Pro-atherogenic effects of EDP in mouse models of atherosclerosis have been already reported [27]. Monocyte migration and reactive oxygen species production in mouse monocytes following EDP stimulation are decreased by DANA. In addition, in LDLR^−/−^ mice devoid of NEU1 in immune cells, decreased plaques size in aortic roots are observed associated with reduced macrophages content. Together with the present study, these results suggest that the pro-atherogenic effects of EDP would start at early stage of atherosclerosis development, by increasing the recruitment of circulating monocytes to inflamed endothelial cells and enhancing their transendothelial migration capacities.

In conclusion, our data provide new important insights on how, by increasing the catalytic (sialidase) activity of NEU1, EDP binding to the ERC would regulate important pathophysiological processes. We identified a common action mechanism by which by stimulating the catalytic activity of the NEU1 subunit associated to the ERC, EDP binding to the ERC induces desialylation of key membrane glycoproteins associated to NEU1 and leads to modulation of cell interactions. By this newly discovered mode of action, new biological functions are anticipated for the ERC, through NEU1, in diseases involving elastic fibers remodeling and degradation. A main issue that remains to be investigated is how EDP binding to the ERC increases NEU1 catalytic activity within the receptor complex. The optimum pH for the lysosomal enzyme is acidic (pH 4.5) unlike the plasma membrane-bound sialidase which has an optimum pH at around 6.5. Therefore, this increased membrane NEU1 sialidase activity following cell stimulation by EDP could not be attributed to the lysosomal pool of this sialidase [42]. Moreover, it has been demonstrated that the EBP subunit of the ERC is never targeted to lysosomes [43–45]. Conformational changes within NEU1 are rather favored but remains to be demonstrated.

## Materials and methods

### Reagents, chemicals and antibodies

Mouse monoclonal anti-ICAM-1 (6.5B5), rabbit polyclonal (H-300) and mouse monoclonal (F-8) anti-NEU1 antibodies were purchased from Santa Cruz Biotechnology. Mouse monoclonal anti-β_2_ integrin antibody (MEM48) and isotype control antibodies were from Merck. Dylight 800-conjugated anti-mouse and rabbit IgG antibodies, and EZLink® sulfo-NHS-LC-biotin were from ThermoScientific. HRP-conjugated anti-mouse and rabbit IgG antibodies were from Cell Signaling. Human Fc Block reagent was purchased from BD Pharmingen. Human ICAM-1 Fc chimera recombinant protein and goat anti-human IgG Fc secondary antibody were from ThermoFisher Scientific. Protein G and Streptavidin sepharose beads were purchased from Sigma and GE Healthcare, respectively. Biotinylated SNA and MALII lectins were purchased from Vector laboratories. Rat collagen type I was from R&D Systems. 2-deoxy-2,3-didehydro-N-acetylneuraminic acid (DANA), protease cocktail inhibitor, Calcein-AM and bovine fibronectin were from Sigma. ThinCert™ Cell Culture Inserts (6 wells, pores of 8 µm) were purchased from Greiner Bio-one. The V14 peptide (VVGSPSAQDEASPL) was synthesized by Genecust with 99% purity.

### Cell lines

The human monocytic THP-1 cell line was cultured at 37 °C and 5% CO_2_ atmosphere in RPMI 1640 medium supplemented with 10% heat-inactivated fetal bovine serum, 100 U/mL penicillin and 0.1 mg/mL streptomycin.

HUVEC were cultured at 37 °C and 5% CO_2_ atmosphere in Endothelial Cell Growth medium (2% FCS, 0.1 ng/mL epidermal growth factor, 1.0 ng/mL basic fibroblast growth factor, 1.0 g/mL hydrocortisone, 0.4% endothelial cell growth supplement/heparin, 50 ng/mL gentamicin, 50 ng/mL amphotericin B), according to the supplier’s recommendations. HUVEC were used between passages 2 and 5.

### Kappa-elastin preparation

EDP were prepared as described previously [25]. Briefly, insoluble elastin was prepared from bovine ligamentum nuchae by hot alkali treatment. Purity was assessed by comparing its amino acid composition to the one predicted from the elastin gene product. Soluble EDP were then obtained from insoluble elastin as described [25]. The obtained mixture of EDP, termed kappa-elastin (κE), has been shown to contain several peptides harboring the bioactive GxxPG motifs [16]. Composition of EDP from κE was compared to EDP obtained after proteolysis of human elastin by neutrophil elastase by mass spectrometry analysis and shown to contain similar peptides harboring the bioactive GxxPG motif [16]. In addition, κE has been shown to exhibit the same biological properties as elastin hydrolysates obtained by human neutrophil elastase [16, 46].

### Co-immunoprecipitations

THP-1 monocytes in RPMI medium were incubated, or not, with κE (50 µg/mL) for 1 h at 37 °C. Then, cells were washed two times with PBS by centrifugation (800* g*, 10 min, 4 °C) and resuspended in 1 mL cold TEM buffer (75 mM Tris, 2 mM EDTA, 12 mM MgCl_2_, protease inhibitor cocktail, 10 mM NaF, 2 mM Na_3_VO_4_, pH 7.5) containing 1% CHAPS. After sonication, lysates were centrifuged at 600* g* for 10 min to remove nuclei and non-lysed cells. Samples were then solubilized during 4 h at 4 °C under gentle end-over-end mixing. After centrifugation at 20,000* g* (45 min, 4 °C), the supernatant was recovered and immunoprecipitations were performed using 4 µg mouse monoclonal anti-NEU1 or 3 µg anti-β_2_ integrin antibodies and protein G Sepharose beads. After washes, immunoprecipitated proteins were eluted with SDS-PAGE loading buffer and subjected to SDS-PAGE and immunoblotting. Immunoblottings were performed using polyclonal rabbit anti-NEU1 (1/500) or mouse monoclonal anti-β_2_ integrin (1/500) antibodies, and immunoreactivity was revealed using HRP-conjugated secondary antibodies (1/10,000) for the co-immunoprecipitated protein, and Dylight 800-conjugated secondary antibodies (1/10,000) for the immunoprecipitated protein. Immunoreactive bands were visualized with the Odyssey Fc scanner (LI-COR). A similar protocol was used for HUVEC except that the day before for experiments, HUVEC were pre-stimulated with PMA (100 nM, overnight) to allow increased expression of adhesive glycoproteins as evidenced here by increased expression of ICAM-1. Co-immunoprecipitations were performed using 4 µg mouse monoclonal anti-NEU1 or anti ICAM-1 antibodies.

### Lectin pull down assay

Lectin pull down was performed on THP-1 cells or PMA-pre-stimulated HUVEC incubated, or not, with κE (50 µg/mL), κE/V14 (molar ratio 1:2) or V14 alone for 1 h at 37 °C. Cells were washed three times in PBS and resuspended in 1 mL cold Tris/NaCl bufer (100 mM Tris, 80 mM NaCl, protease inhibitor cocktail, 10 mM NaF, 2 mM Na_3_VO_4_, pH 8) without detergent. After sonication, lysates were centrifuged at 600* g* for 10 min to remove nuclei and non-lysed cells. Then, crude membranes were pelleted by centrifugation at 20,000* g* during 45 min at 4 °C. After solubilization in Tris/NaCl buffer containing 1% NP-40 for 3 h at 4 °C, samples were centrifuged at 20,000* g* (45 min, 4 °C) and the supernatant (solubilized crude membrane proteins) was recovered. For each condition, equal amounts of membrane proteins were incubated with 50 μg/mL biotinylated SNA or MALII lectins (overnight, 4 °C). Streptavidin agarose beads were then added for 1 h at 4 °C. The beads were washed once with TBS/1% Triton X-100 and twice with TBS/0.5% Triton X-100, and directly resuspended in SDS-PAGE loading buffer, boiled and subjected to SDS-PAGE and immunoblotting. Western blots were performed using mouse monoclonal anti-β_2_ integrin (1/500) or ICAM-1 (1/500) antibodies and immunoreactivity was revealed using HRP-conjugated anti-mouse antibodies (1/10,000) and visualized with the Odyssey Fc scanner (LI-COR).

### Biotinylation of cell surface proteins

THP-1 cells in suspension and adherent HUVEC grown in 10 cm plates pre-stimulated with 100 nM PMA (overnight, 37 °C) were used. Cells were washed three times in PBS, incubated with 0.5 mg/mL of EZ-Link® sulfo-NHS-LC-biotin for 30 min at 4 °C, and quenched with 100 mM glycine (30 min, 4 °C). Cells were then scraped in TEM buffer (75 mM Tris, 2 mM EDTA, 12 mM MgCl_2_, protease inhibitor cocktail, 10 mM NaF, 2 mM Na_3_VO_4_, pH 7.5) containing 1% Triton X-100, sonicated and incubated under gentle end-over-end mixing (3 h, 4 °C) for solubilization of membrane proteins. Lysates were centrifuged (20,000* g*, 45 min, 4 °C) to pellet insoluble material and supernatants were incubated with 25 μL streptavidin agarose beads for 45 min at 4 °C to purify biotinylated membrane proteins. After several washes, biotinylated proteins were eluted from the beads by Leammli buffer and subjected to SDS-PAGE and immunoblotting. Western blots were performed using mouse monoclonal anti-β_2_ integrin (1/500) or ICAM-1 (1/500) antibodies and immunoreactivity was revealed using HRP-conjugated anti-mouse antibodies (1/10,000) and visualized with the Odyssey Fc scanner (LI-COR).

### Monocyte adhesion to HUVEC

HUVEC (between passages 2 and 5) were seeded on 24-well plates and cultured for 2 days at 37 °C and 5% CO_2_ atmosphere to form a confluent monolayer. The day before experiment, HUVEC were stimulated with 100 nM PMA (overnight). THP-1 monocytes in RPMI complete medium were adjusted to 1.10^6^/mL and stimulated, or not, with κE (50 µg/mL, 1 h). Monocytes were then labelled with 2.5 µM Calcein-AM (30 min). After 2 washes with hot RPMI complete medium, calcein AM-labeled monocytes were adjusted to 7 × 10^5^/mL and 3.5 × 10^5^ cells (500 µL) were added per well on confluent HUVEC monolayer and incubated for 30 min (37 °C, 5% CO_2_ atmosphere). Non-adherent monocytes were gently washed off using hot PBS and adherent monocytes were fixed with 4% paraformaldehyde in PBS for 15 min. After 2 washes with PBS, monocyte adhesion was visualized under an invert fluorescent microscope (AXIO, Zeiss). The percentage of surface area covered by fluorescent monocytes was then calculated using the “%Area” parameter of ImageJ software, after manual threshold, from ten different fields per well. Each experiment was performed in duplicate. When indicated, THP-1 monocytes were incubated with DANA (400 µM), anti-β_2_ integrin blocking antibody (10 µg/mL) or control isotype (10 µg/mL) for 1 h at 37 °C before being stimulated by κE (50 µg/mL), V14 + κE (molar ratio 2:1) or V14 peptide alone for 1 additional hour. For HUVEC, and when indicated, cells were incubated with DANA (400 µM) for 1 h at 37 °C before being stimulated by κE (50 µg/mL), V14 + κE (molar ratio 2:1) or V14 peptide alone for 1 additional hour.

### Monocyte transendothelial migration

Monocyte transendothelial migration was assessed using transwells. Inserts were coated with 7 µg/mL fibronectin for 1 h at 37 °C before seeding HUVEC (between passages 2 and 5) in the upper chamber of each transwell, and grown for 2 days to form a confluent monolayer. The day before experiment, HUVEC were stimulated with 100 nM PMA (overnight, 37 °C). The day of experiment, and when indicated, HUVEC were incubated with DANA (400 µM) for 1 h at 37 °C before being stimulated by κE (50 µg/mL), V14 + κE (molar ratio 2:1) or V14 peptide alone for 1 additional hour. For THP-1 monocytes, cells were stimulated or not by κE (50 µg/mL) for 1 h at 37 °C. Calcein AM-labeled THP1 cells (2.5 × 10^5^ cells) were added to the upper chamber and allowed to migrate through the HUVEC monolayer into the lower chamber for 2 h (37 °C, 5% CO_2_ atmosphere). Subsequently, the upper chamber was removed to stop transmigration. The fluorescence intensity in the lower compartment was measured in black 96-wells microplate using TECAN infinite F200 Pro microplate reader and excitation and emission filters at 490 and 520 nm, respectively.

### Confocal microscopy

THP-1 monocytes and HUVEC adherent to sterile coverslips were used. To allow THP-1 to adhere to coverslips, coverslips were coated with rat collagen. Cells were stimulated, or not, with κE (50 µg/mL, 1 h), then washed three times with PBS before fixation with 4% paraformaldehyde in PBS for 15 min and permeabilization by 0.2% Triton X-100 in PBS for 10 min. After blocking with 10% goat serum in PBS for 1 h, cells were incubated with mouse monoclonal anti-β_2_ integrin (5 µg/mL), mouse monoclonal anti-ICAM-1 (5 µg/mL) and/or rabbit polyclonal anti-NEU1 (4 µg/mL) in PBS containing 1% BSA overnight at 4 °C. Coverslips were then washed three times with PBS and incubated with Alexa Fluor 488-conjugated goat anti-mouse and Alexa Fluor 568-conjugated goat anti-rabbit antibodies (1/1000) in PBS containing 1% BSA for 45 min at room temperature. Coverslips were mounted, visualized with a laser scanning microscope (LSM 710 NLO, Zeiss) and analyzed by ImageJ software using the Colocalization plugin.

### Flow cytometry

To assess the ability of ICAM-1 to bind to κE-stimulated THP-1 monocytes, a flow cytometry assay was used, as previously described [14]. Recombinant human ICAM-1-Fc fusion protein (25 μg/mL) was incubated with PE-conjugated anti-Fc antibodies (25 µg/mL) at 4 °C overnight before use. THP-1 monocytes in RPMI complete medium (1 × 10^6^/mL per condition) were stimulated, or not, with κE (50 µg/mL, 1 h) and centrifuged at 500 g for 5 min. After washing in FluoroBrite DMEM media containing 0.5% BSA, cells were resuspended in FluoroBrite DMEM media containing 0.5% BSA and 2 µg human Fc block reagent to block endogenous Fc receptors (15 min, room temperature). Cells were then centrifuged and incubated with the above ICAM-1-Fc/PE-conjugated anti-Fc antibodies complexes for 30 min at room temperature. After washing in FluoroBrite DMEM media containing 0.5% BSA, cells were resuspended in PBS and analyzed with the BD Accuri C6 flow cytometer equipped with 585/40 nm band pass filter on FL2 (excitation 488 nm) to collect PE signal. Acquisition and processing data from 20,000 cells were performed and analyzed using BD CSampler Software.

### Statistical analysis

Results were expressed as mean ± SEM. Statistical significance was evaluated using unpaired Student’s *t* test, and *p* values of less than 0.05 were considered as statistically significant.

## Data Availability

All data generated or analyzed during this study are included in this published article.

## Abbreviations

DANA: 2-Deoxy-2,3-didehydro *N*-acetylneuraminic acid
ECM: Extracellular matrix
EDP: Elastin-derived peptides
ERC: Elastin receptor complex
HUVEC: Human umbilical vein endothelial cells
ICAM-1: Intercellular cell adhesion molecule-1
JAM: Junctional adhesion molecule
κE: Kappa-elastin
NEU1: Neuraminidase-1
MALII: *Maackia amurensis* Lectin II
PECAM-1: Platelet endothelial cell adhesion molecule-1
PMA: Phorbol myristate acetate
PPCA: Protective protein/cathepsin A
SNA: *Sambucus nigra* Agglutinin
